# Cognitive Impairment and Risk Factors of Alzheimer's Disease Among Elderly PeopleLiving in the Geriatric Home in Coimbatore Population, India

**DOI:** 10.1002/alz70855_099236

**Published:** 2025-12-23

**Authors:** Gomathi Mohan, Kanagadharshini Subramani, Kabilan Malaiyarasan

**Affiliations:** ^1^ Avinashilingam Institute for Home Science & Higher Education for Women, Coimbatore, Tamil Nadu, India; ^2^ Karpagam Academy of Higher Education, Coimbatore, Tamil Nadu, India

## Abstract

**Background:**

Cognitive impairment and dementia are devastating health concerns for the elderly population. As cognitive abilities decline, individuals with dementia may struggle with basic activities of daily living such as dressing, bathing, cooking, and managing finances. Dementia can be caused by various underlying diseases, with Alzheimer's disease (AD) being the most common disease. In India, the estimated age‐standardized dementia prevalence for people aged ≥ 60 years is eight percent (8.8 million individuals). The main aim of this study was to screen the cognition, behaviour, and risks of Alzheimer's Disease in elderly patients living in a geriatric home in the Coimbatore population, India.

**Method:**

Cognitive impairment in elderly patients (*N* = 64) was evaluated using the mini‐mental state examination and mini‐cognitive assessment tools. Risk factors for AD were predicted using demographic risks, and a geriatric home‐based study was conducted at a geriatric home, Coimbatore, from November 2023 to December 2024. Data were collected using a structured questionnaire consisting of sociodemographic variables, the NPI scale, the Mini‐Mental State Examination, and Mini Cognitive Assessment tools.

**Result:**

In this study, 88 participants were involved; however, 64 completed the survey. Among the older participants screened for cognitive impairment, 46% were positive for probable AD. The prevalence of older participants who screened positively for cognitive impairment was high in geriatric homes in Coimbatore District, India. The independent predictors of AD among these individuals were older age and poor family or social support. APOE4 genotypic risk assessment showed that the rs429358 polymorphism in 8 patients was a major risk factor for AD.

**Conclusion:**

Therefore, research on the APOE genotype is essential for identifying the genetic risk of AD. Therefore, we recommend providing strong social support and screening for APOE4 risk alleles among elderly people aged 60 years to manage the global health burden.

